# Efficacy of stem cells in bone rehabilitation in patients with alveolar bone atrophy: a systematic review

**DOI:** 10.4317/medoral.25667

**Published:** 2022-12-24

**Authors:** Martín Pérez-Leal, Marta Scanferla, María Carmen Carceller, Nicla Flacco

**Affiliations:** 1 Universidad Europea de Valencia, Faculty of Health Sciences, Department of Dentistry, Valencia, España

## Abstract

**Background:**

Biomedical engineering proposes the use of stem cells as a bone rehabilitation treatment in patients with alveolar bone defects. Many authors suggest that this innovative technique could represent the future of bone regeneration in dentistry. The present study systematically reviewed the efficacy of stem cells in bone regeneration in patients with alveolar bone atrophy.

**Material and Methods:**

The study was developed following the criteria of the PRISMA guideline (2020). The literature review was conducted in Pubmed, Medline Complete, and Scopus. The search algorithms used the following key words: stem cells, bone regeneration, and alveolar ridge augmentation. To assess the risk of bias, the CASPe methodology was used.

**Results:**

Seven clinical trials in humans were included in this systematic review. In all the studies, the proposed objective of bone regeneration by using stem cells was achieved, although in a different way with different results. Although the authors of the analysed clinical trials achieved favourable results, they highlighted the presence of multiple limitations throughout bone regeneration treatments, such as scarce scientific literature on stem cells, a reduced number of follow-up studies, and a lack of a standardized international protocol.

**Conclusions:**

Based on the analysed studies, it is concluded that the therapy proposed by tissue engineering through the use of stem cells to rehabilitate patients with bone atrophies can be considered effective. In addition, the need for further studies and standardization of protocols is highlighted.

** Key words:**Stem cells, bone rehabilitation, alveolar bone atrophy, tissue engineering.

## Introduction

Alveolar bone remodelling is defined as a physiological process characterized by a constant balance between bone resorption and bone formation. Any factor, such as trauma or periodontal disease, can alter this process. In fact, to guarantee daily functions such as chewing, it is necessary to regenerate the alveolar bone to place, secondly, dental implants (1). Almost 2.2 million bone regeneration surgeries are estimated each year in the world, making the bone one of the most transplanted tissue. As an alternative to the classic Gold Standard graft interventions, characterized by multiple complications such as the need for a second surgical area, long intervention time, and risk of nerve damage during bone extraction, the tissue engineer proposes bone rehabilitation using stem cells. The latter have high capacities for self-regeneration, differentiation in several cell lines and are found throughout the body, such as in the umbilical cord, bone marrow, and teeth (2).

Under sTable conditions, bone mass and density are determined by receptor activator of NF-κB (RANK), receptor activator of NF-κB ligand (RANKL) and osteoprotegerin (OPG); bone remodelling occurs by the continuous RANK-RANKL-OPG regulatory process. The binding of the ligand to its receptor (RANKL to RANK) favours the differentiation of preosteoclasts into mature osteoclasts. OPG protects bone from excessive resorption by binding to RANKL and preventing it from binding to RANK. Thus, the relative concentration of RANKL and OPG in bone is a major determinant of bone mass and strength (3). The RANKL-RANK-OPG process must always be kept in balance throughout life, favouring tissue homeostasis. Otherwise, a pathological process is triggered characterized by RANKL hyperactivity and OPG hypoactivity, obtaining excessive bone resorption that leads to the loss of bone levels.

Stem cells, the object of study in biomedical engineering, are defined by the scientific community as undifferentiated, immature cells, with self-renewal capabilities and the generation of different types of cells. Embryonic stem cells (ESCs) and adult stem cells (ASCs) are distinguished. ESCs are present only in the embryonic stage and generate any type of body cell, as they can transform into any of the three embryonic lines: endoderm, mesoderm, and ectoderm. ASCs are present in mature tissues and the umbilical cord. These are multipotent and unipotent cells that derive from the differentiation in any of the three embryonic lines and they are irreversible. They perform an important function in the maintenance and restoration of the tissue of the organ in which they are found. Four types of stem cells have been identified: totipotent, pluripotent, multipotent, and unipotent. The totipotent cell derives from the fusion of the male gamete with the female, present only in the embryonic stage. This type of cell gives rise to an organism, it has the capacity to differentiate into embryonic tissues, such as the ectoderm, or extra-embryonic tissues, for example, the placenta. The pluripotent cell has differentiation capacity in any of the three embryonic lines but cannot give rise to an organism. The multipotent stem cell, known as organ-specific stem cell, is capable of giving life to organs and is located in various parts of the human body. Unipotent cells, defined as oligopotent cells, differentiate into a single cell line. In daily practice, the most used type of stem cells is adult ones. Embryonic cells are used little due to ethical debate and legal complications, related to damage in human embryos. There are also other motivations that favour the implementation of ASC over ESC such as the low percentage of developing tumours or post-rehabilitation rejection. (4,5)

Thus, this systematic review aimed to collect and summarize the information about the efficacy of stem cells in bone regeneration in patients with alveolar bone atrophy.

Abreviations: NF-κB; nuclear factor kappa B, RANK; receptor activator of NF-κB, RANKL; receptor activator of NF-κB ligand, OPG; osteoprotegerin, ESC; embryonic stem cells, ASC; adult stem cells.

## Material and Methods

This systematic review was conducted following the PRISMA statement (2020), Preferred Reporting Items for Systematic Reviews and Meta-Analyses (6). The study was registered in PROSPERO under registration number CRD42022322548. The following focus question was employed according to the population, intervention, comparison, and outcome (PICO) study design (7): Is stem cell therapy an effective strategy to promote bone regeneration in patients with alveolar bone atrophy? P: Patients with alveolar bone atrophy, I: Intervention with stem cells to regenerate bone, C: Compared with other classic techniques, O: Effectiveness of stem cells technique in bone regeneration. To carry out this study, only clinical studies in humans published in English, Spanish and Italian between January 2002 and April 2022, in scientific databases: Pubmed, Medline Complete, and Scopus were analysed. The Boolean search algorithms applied in the three databases used the following key words: stem cells, bone regeneration, and alveolar ridge augmentation. The literature search was conducted using the combinations of the following Medical Subject Heading (MeSH) and text words: ("stem cells"[MeSH Terms] OR ("stem"[All Fields] AND "cells"[All Fields]) OR "stem cells"[All Fields]) AND ("bone regeneration"[MeSH Terms] OR ("bone"[All Fields] AND "regeneration"[All Fields]) OR "bone regeneration"[All Fields]) AND ("alveolar ridge augmentation"[MeSH Terms] OR ("alveolar"[All Fields] AND "ridge"[All Fields] AND "augmentation"[All Fields]) OR "alveolar ridge augmentation"[All Fields]). Additional relevant articles were searched manually to not exclude any publication of interest. All studies selected for this systematic review had to meet the eligibility criteria. The inclusion criteria took into consideration clinical studies on humans whose objective of the study was the use of stem cells in bone regeneration in bone defects in patients with alveolar bone atrophy. The exclusion criteria were: bibliographic reviews, systematic reviews and meta-analyses, animal or *in vitro* studies, and incomplete studies where all the parts that make up a scientific article are missing. Those papers in which bone regeneration by stem cells in patients with alveolar bone atrophy was not indicated or studied were also excluded. The evaluation of risk of bias was assessed through the Critical Appraisal Skills Program CASPe methodology, and the following criteria were considered: the presence of a specific topic, such as rehabilitation of alveolar bone atrophies, the relevance of the method used to answer the question of interest, description of the relationship with the objective of the research and usefulness of the results, considering the reproducibility of the study.

## Results

**Figure 1:**
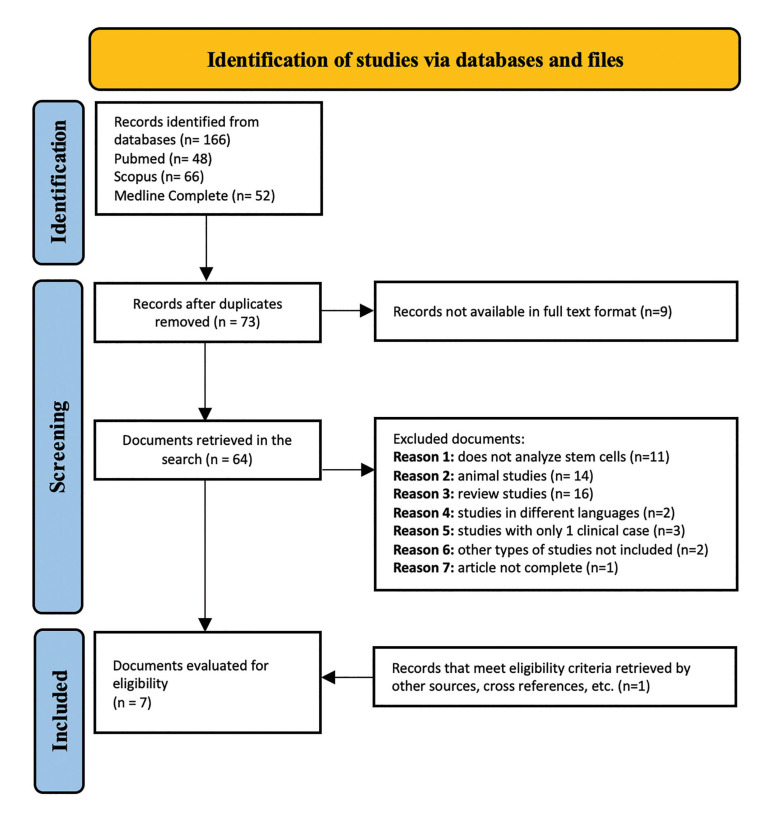
Flow Chart following the PRISMA statement (2020), in which the study selection process and results of the bibliographic search for this systematic review are graphically represented, including reasons for excluding some of the studies.

**Figure 2:**
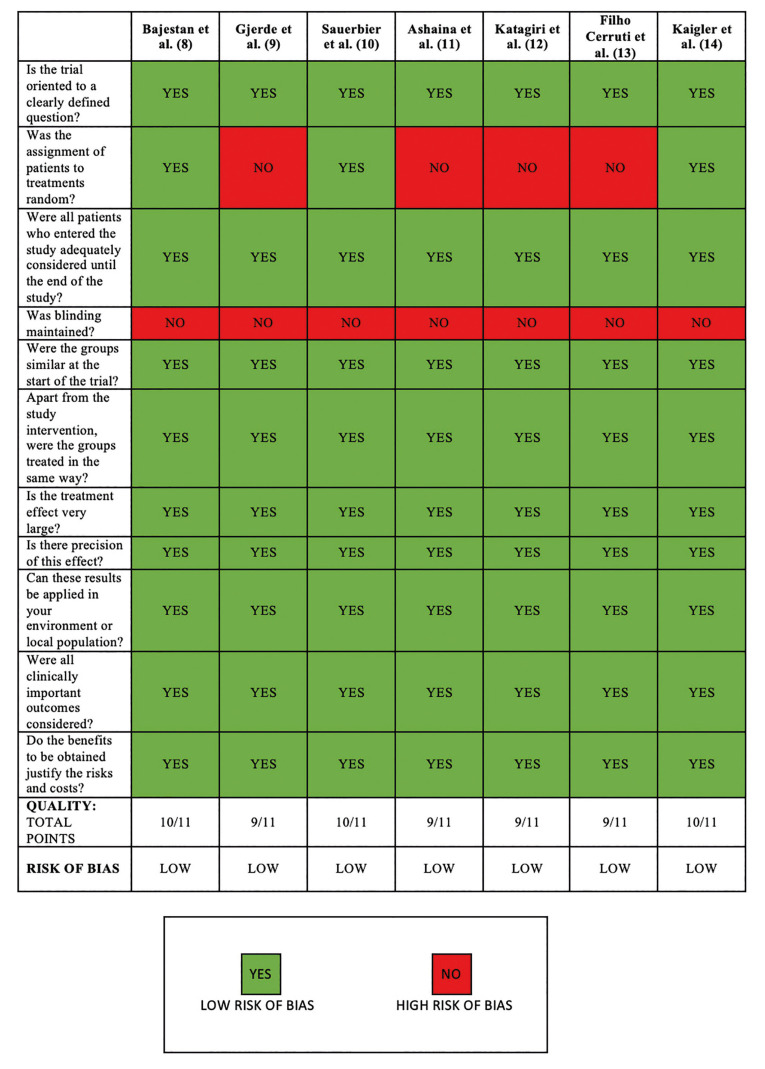
Evaluation of methodological quality and risk of bias using the CASPe methodology.

The total number of articles found in the three databases was 166. After removing duplicates, 9 more papers were removed because they were not recoverable and the rest of the studies (*n*=64) were screened. 58 were excluded because they did not meet the eligibility criteria (humans, clinical studies, and regeneration with stem cells), and others were reviews written in other languages or not complete. One publication, which met the eligibility criteria, was retrieved through other sources and cross-references. Finally, 7 clinical trials were included (Fig. 1) and analysed with their respective variables: study group, type of treatment, age, sex, cell source, and type of study (Table 1). A critical reading of the studies was carried out using the CASPe methodology. All the articles were classified as LOW risk of bias (Fig. 2).

**Table 1:**
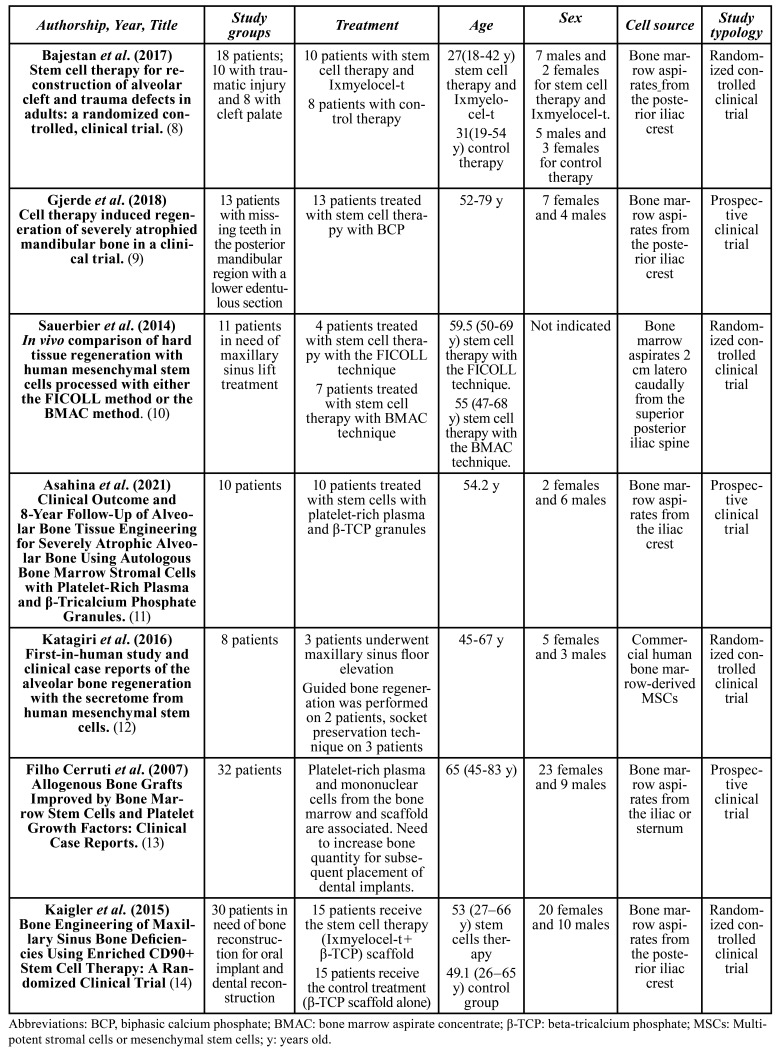
Data extracted from the analysed studies were classified according to their respective variables: study group, type of treatment, age, sex, cell source, and type of study.

In the selected studies, a total of 122 patients (between treated and controls) were included, in an age range between 18 and 83 years, in prospective clinical trials and randomized controlled clinical trials. Considerable heterogeneity was observed in the seven clinical trials regarding the treatment, study design, evaluation period, and study population.

Bajestan *et al*. (8) examined the safety and efficacy of expanded stem cell-based therapies to regenerate alveolar bone in patients with alveolar bone defects and to determine whether Ixmyelocel-t therapy could regenerate bone and long-term stability for subsequent implant placement. They selected 18 patients with horizontal alveolar crest atrophy, 8 due to clefts, and 10 due to trauma. The age was between 27-31 years; both sexes were included. The patients were divided into two groups: 8 patients were randomly assigned to the control group (autogenous block grafting) and another 10 patients to the bone regeneration therapy group with Ixmyelocel-t (stem cell therapy group). On the day of the intervention, both types of intervention were performed under local anaesthesia, and regenerative material was placed in the bone defect. 4 months later dental implants were placed and at 6 months stability was assessed. Implants were placed in all 8 patients in the control group and only in 5 of 10 in the stem cell bone regeneration group. Patients excluded at the time for implant placement undergo another bone regeneration procedure and were evaluated 4 months later. In the control group one implant failed to achieve osseointegration and was excluded from the study. Success was achieved in 17 treatments out of 18. The result was more satisfactory in patients with bone defects due to trauma compared to those with cleft palate both in the control group or stem cell therapy. Gjerde *et al*. (9) aimed to assess the degree of bone regeneration with mesenchymal stem cells (MSCs) of bone marrow origin associated with biphasic calcium phosphate (BCP) in severe resorption of the mandibular alveolar crest. 13 patients were selected, aged between 52 and 79 years. For the intervention, autologous cells were placed at the site of atrophy collected from the posterior superior iliac crest and treated in the laboratory combined with beta-tricalcium phosphate (β-TCP). Titanium-reinforced non-resorbable polytetrafluoroethylene (PTFE) membranes were fixed to the bone with microscrews. Placement of dental implants was performed, and after 2-4 weeks the crowns were screwed. Follow-up control was conducted between 1.2 and 4 months after the day of the intervention. A successful outcome was achieved in 11 of 13 patients. Two patients were excluded due to insufficient bone marrow cells. Sauerbier *et al*. (10), wanted to compare new bone formation in maxillary sinus augmentation interventions using biomaterial associated with MSCs separated by two different isolation methods: the synthetic polysaccharide (FICOLL) method (control group) or the bone marrow aspirate concentrate (BMAC) method (test group). The FICOLL open method, a valid treatment for the collection of Human Mononuclear Cells (MNC), has limitations such as handling time and the need for a manufacturing laboratory. So, the BMAC closed system, which can be applied in health centres without the possibility of a manufacturing laboratory and at a lower cost, was proposed. 11 candidates were included, 4 for the FICOLL technique with a mean age of 59.5 years and 7 for the BMAC technique with a mean age of 55 years. Stem cell collection was performed at the level of the posterior superior iliac spine and manipulation in the laboratory for FICOLL and in the operating room for BMAC. Within three months of cell placement at the atrophic site, dental implants were placed under local anaesthesia (17 FICOLL and 33 BMAC). All patients participated until the end of the study. In all cases, good recovery after the surgical procedure was identified. Similar results were obtained with FICOLL and BMAC. It is estimated that the efficacy of the BMAC technique was 4.6% higher than that of FICOLL. A follow-up period of 2 years was carried out. No rejection of any of the 17 implants in the FICOLL group was verified, unlike the BMAC group, where 1 implant out of 33 turned out to be unsuccessful. After placement, no loss of implants was detected. Both groups obtained excellent results. Asahina *et al*. (11) examined the feasibility, safety, and efficacy of bone tissue engineering in patients with atrophic alveolar bone using bone marrow-derived MSCs. The clinical status was assessed 8 years later and the potential problems of regenerating bone using stem cells were identified. 10 patients presenting a need for sinus floor elevation or alveolar crest augmentation were selected. Bone marrow stem cells were aspirated from the iliac bone crest under local anaesthesia. On the day of transplantation, a mixture of platelet-rich plasma (PRP) growth factors with stem cells, autologous thrombin, 10% CaCl2, and β-TCP was placed at the site of atrophy. At 6 months the dental implants were placed. Successful positive long-term follow-up was conducted for 5 of 8 subjects. Katagiri *et al*. (12) intended to evaluate the safety and use of MSC culture conditioned medium (MSC-CM) for alveolar bone regeneration in patients who need bone augmentation before placing dental implants. Eight patients were included, aged between 45-67 years, partially edentulous in need of bone augmentation, including maxillary sinus floor elevation, guided bone regeneration, and socket preservation. The authors chose the option of buying human MSCs and culturing them. During the treatment, MSC-CM was mixed with β-TCP. In 5 cases, implants were placed simultaneously with the bone augmentation process, and in 2 cases at a distance of 8-9 months. No complications were identified and initial stability was achieved. β-TCP is widely used for its excellent osteoconductivity but resorbs over a long period. Indeed, in this study, β-TCP mixed with MSC-CM promoted early resorption and replacement of new bone compared to β-TCP without MSC-CM. Filho Cerruti *et al*. (13) wanted to describe a tissue regeneration technique using allogeneic bone obtained from reliable bone banks in combination with autologous mononuclear cells (MNC) isolated from patients’ iliac crest or sternum and growth factors like platelet-rich plasma (PRP). They claimed to demonstrate short-term and long-term clinical results. 32 patients were included, 23 women between 45-83 years old and 9 men between 58-75 years old. They underwent considerable bone grafting treatment and subsequent placement of dental implants. The areas of surgical intervention were anterior and posterior maxilla. The bone graft alveolar crest augmentation and sinus elevation were performed using a mixture of particulated excess of bone graft, PRP, thrombin, and CaCl2 which was used as a bedding for the bone grafts. The same day of intervention, dental implants were also placed. Success was achieved in 30 out of 32 bone graft interventions, which determined correct osseointegration and a sufficient amount of bone to place dental implants. In all clinical cases, the amount of bone obtained after bone regeneration treatment is sufficient in height and width to place dental implants. Between 2 and 4 years later, another review was performed to assess the clinical condition, and no bone loss was detected. Kaigler *et al*. (14) demonstrates how stem cell therapy can be considered effective for the treatment of different types of bone defects at the oral and craniofacial level, complex or combined with other treatment modalities, in which accelerated bone healing and viable bone are desired. 30 patients with severe bone atrophy of the upper jaw and in need of bone reconstruction for an oral implant and dental reconstruction were recruited to participate in this randomized, controlled clinical trial. 15 patients were randomized to receive the stem cell therapy (Ixmyelocel-t + β-TCP) scaffold, 10 females and 5 males between 27-66 years old. 15 patients received the control treatment (β-TCP scaffold alone), 10 females and 5 males between 26-65 years old. Each subject only received one of the two possible treatments. 4 withdrew from the study before undergoing any treatment-related procedures. Of the 26 participants, 2 dropped out before study completion. The clinical procedures were no different between treatment groups, and in both groups, favourable function and esthetics were achieved with the final tooth restorations. The clinical surgical parameters of the two treatments were equivalent between groups. There was one graft failure in the treatment group and one implant failure in the control group. No serious adverse events were reported in any of the above-mentioned studies.

## Discussion

Alveolar bone rehabilitation treatment using stem cells has great potential and shortly could have high success rates to replace gold standard surgical interventions. Although bone tissue engineering in dentistry has been studied for many years, a limited number of long-term studies are published in the scientific literature and a standardized protocol has not yet been established. In addition, this technique presents some difficulties. Bajestan *et al*. (8) reported the variability of the methods of isolation and expansion of cell populations. Gjerde *et al*. (9) informed about the morbidity of the donor, the limited amount of bone to be reconstructed, and the eventual unpredicTable resorption of the graft. Sauerbier *et al*. (10) considered how the FICOLL method, unlike BMAC, has greater limitations since, being an open system, it involves the need to leave the operating room to process the cells in a laboratory, and it has a high cost. The use of the materials in association with stem cells in this study is different from the studies conducted by Bajestan *et al*. (8) and Gjerde *et al*. (9).

Unlike the limitations of previously conducted studies, Asahina *et al*. (11) stated that their study may be limited by factors such as the anatomical environment and the surgical procedure. Katagiri *et al*. (12) share some limitations also stated by Bajestan *et al*. (8) and Sauerbier *et al*. (10), such as the use of adult stem cells, high cost, and strict regulation by the authorities on the manipulation of these cells. Filho Cerruti *et al*. (13) achieve optimal results as well as the studies carried out by Gjerde *et al*. (9) and Asahina *et al*. (11). At the same time, the authors indicated limitations due to the scarce scientific evidence on the regenerative advantages and disadvantages of PRP and its relative use in bone grafts.

Kaigler *et al*. (14) consider the source of cells and the cell expansion protocol a key to the success, development, and continuous optimization of this type of regenerative treatment. The same authors state that despite recent advances in the field of tissue engineering and regenerative medicine, the reconstruction of large bone defects has always been treated using autogenous grafts, allografts, xenografts, and synthetic alloplastic materials. Due to the multiple disadvantages that this type of treatment provides, the article indicates the need to develop more specific cell and tissue therapies to overcome the limitations of traditional treatments. They note how stem cell therapy can be defined as a promising tissue engineering strategy to improve tissue regeneration and promote the formation of both hard and soft tissues. The main limitation found is related to the limited typology of the cell populations used for therapy, as indicated by Bajestan *et al*. (8), Sauerbier *et al*. (10), and Katagiri *et al*. (12).

Regarding the origin of the stem cells, most authors such as Bajestan *et al*. (8), Gjerde *et al*. (9), Ashaina *et al*. (11) and Kaigler *et al*.(14), agree on the extraction of the cells from the iliac area, specifically from the iliac crest. However, Sauerbier *et al*. (10) obtain the cells from the iliac spine, and in the study of Filho Cerruti *et al*. (13) the iliac zone is not specified, in addition they also propose the sternum. On the other hand, Katagiri *et al*. (12) suggest the use of commercial human bone marrow-derived MSCs to carry out tissue regeneration trials in patients.

Limitations have been found in the search for scientific articles, given that the object of study is relatively new. Despite the large number of scientific papers found at the beginning of the search, after reading the abstract or full text, and selecting articles according to the search objectives and inclusion criteria, the included studies are few. The topic studied needs a greater number of studies, more in-depth, long-term, and on a larger population. Undoubtedly, scientific research on the implementation of stem cells is limited since an official protocol has not yet been developed by the scientific community, which defines the types of stem cells and associated materials to be used. Ethics and religion play a fundamental role in future scientific advances in stem cell therapy since there is controversy about whether the use of totipotent stem cells, present only in the embryonic period, would be affecting the development of a fetus.

## Conclusions

Based on the analyses carried out in this study, it can be stated that the innovative therapy developed by tissue engineering through the use of stem cells to promote rehabilitation in patients with bone atrophies is effective. The ability of stem cells to regenerate bone can be confirmed and stem cell treatments can be considered a valid alternative to the Gold Standard technique using grafts. However, there is still no common protocol for this type of therapy, nor valid techniques of regeneration through experimentally proven tissue engineering. Currently, there are different proposals for the manipulation of stem cells based on different methodologies, associated with multiple materials. This work highlights the need for more advanced and specific clinical studies in humans based on clinical needs, as well as the standardization of methodology.
